# A Comparison of Single Dose Remimazolam With Dexmedetomidine for the Prevention of Emergence Delirium in Children Undergoing Tonsillectomy and Adenoidectomy Surgery Under Sevoflurane Anesthesia: A Randomized Clinical Trial

**DOI:** 10.1155/anrp/7780635

**Published:** 2025-09-14

**Authors:** Ting Liu, Jing Zhou, Xi-Xi Wang, Si-Fei Gan, Jie-Qiong Liu, Peng-Fei Zhu, Mei-Hong Li, Fang Luo

**Affiliations:** Department of Anesthesiology and Pain Medicine, Hubei Key Laboratory of Geriatric Anesthesia and Perioperative Brain Health, and Wuhan Clinical Research Center for Geriatric Anesthesia, Tongji Hospital, Tongji Medical College, Huazhong University of Science and Technology, Wuhan, China

**Keywords:** dexmedetomidine, emergence delirium, pediatric anesthesia, preschool age, remimazolam

## Abstract

**Introduction:** Emergence delirium, characterized by early postoperative behavioral changes in pediatric patients, poses potential risks to patient safety, resulting in extended hospital stays and increased medical costs. Remimazolam has a rapid onset, moderate half-life, and lower compression on respiratory and circulatory function. This double-blind randomized study aims to compare the incidence of emergence delirium in pediatric patients who received a single dose of remimazolam or dexmedetomidine before the end of sevoflurane anesthesia.

**Methods:** A total of 110 pediatric patients aged 2–12 years, American Society of Anesthesiologists (ASA) Class I or II, undergoing elective tonsillectomy and adenoidectomy were included in this study and randomized into the dexmedetomidine group and remimazolam group (R group) (*n* = 55). Inhalation of sevoflurane was stopped 15 min before surgery after asking the surgeon's opinion, and either 0.2 mg/kg of remimazolam or 0.2 μg/kg of dexmedetomidine was administered. The main and secondary results of the research were both analyzed with the intention-to-treat analysis. The main outcome observed in this study was the incidence of emergence delirium in both groups. Secondary outcomes were vital signs at various time points after administration, Pediatric Anesthesia Emergence Delirium (PAED) scale score for delirium, extubation time, the length of time in the postanesthesia care unit (PACU), postoperative adverse events, and parental satisfaction.

**Results:** The intention-to-treat analysis indicated that the mean age of patients was 5.7 ± 0.4 years, with 62 (56.4%) of them being male. The incidence of emergence delirium was 25.5% overall, with no significant difference seen between the two groups. Compared to the R group, dexmedetomidine decreased the heart rate significantly (*p* < 0.001). There were no differences in extubation time, PACU stay, postoperative adverse events, and parental satisfaction between the two groups. In addition, age is an independent risk factor contributing to the emergence delirium.

**Conclusions:** Discontinuing the inhalation of sevoflurane 15 min before the end of the procedure and administering 0.2 mg/kg of remimazolam intravenously did not show superiority over 0.2 μg/kg of dexmedetomidine in preventing emergence delirium.

**Trial Registration:** Chinese Registry of Clinical Trials: ChiCTR2300072526

## 1. Introduction

Emergence delirium (ED) is an early postoperative behavior change during the recovery period, characterized by symptoms such as crying, thrashing, and disorientation [1]. Based on the use of inhaled agents, the incidence of ED in pediatric anesthetics may reach 40% and is greatest in children aged 2–6 years [2]. Considerable scholarly investigation has been dedicated to the identification of predisposing factors associated with ED. These factors encompass a range of variables such as volatile anesthetics, preschool-aged children, pain, ophthalmology, and otorhinolaryngology procedures, as well as preoperative anxiety [1, 3]. Physicians and hospitals attach importance to the impact of ED, which encompasses potential patient injury, prolonged postanesthesia care unit (PACU) durations, and subsequent maladaptive outcomes such as sleep disruptions, nocturnal enuresis, behavioral outbursts, attention-seeking behaviors, and feelings of isolation [4]. However, the underlying pathogenesis of ED remains unclear. The independent risk factor was thought to be the hasty waking from anesthesia [5]. In contrast to intravenous anesthetics, inhalation anesthetics possess reduced blood solubility and facilitate rapid emergence from anesthesia, thereby increasing the likelihood of ED [6].

Dexmedetomidine, a selective *α*_2_-adrenoceptor agonist, exerts dual pharmacological effects through binding to receptors in the locus coeruleus (mediating superior sedation and anxiolysis) and spinal cord (providing dose-dependent analgesia) [7]. A Bayesian network meta-analysis demonstrated its superior efficacy in preventing ED compared to alternatives including propofol, midazolam, sufentanil, ketamine, and fentanyl [8]. Clinically, intranasal administration (1 μg/kg) shows equivalent prevention to 0.2 mg/kg midazolam [9], while intravenous bolus (5 μg/kg) during induction reduces ED incidence and improves recovery quality in sevoflurane-anesthetized tonsillectomy patients [10]. Although effective when administered as a single dose prior to extubation for reducing postoperative agitation, cough, and shivering, its clinical utility is limited by a prolonged onset time (10–15 min) and cardiovascular effects (dose-dependent bradycardia and hypotension) that require careful infusion rate control [11]. These pharmacokinetic characteristics make it suboptimal as a last-minute rescue medication despite its established prophylactic benefits.

Remimazolam, a benzodiazepine characterized by its ultra-short half-life and specific esterase metabolism, exhibits a swift onset of action [12–14]. Following administration, it demonstrates a rapid onset, with a terminal half-life of a mere 0.75 h in healthy adults [15]. Importantly, the pharmacokinetics of remimazolam follow a three-compartment model in both adults and children [16, 17], with a higher clearance rate observed in children [16]. In comparison to dexmedetomidine, remimazolam exhibits a lower incidence of hypotension and bradycardia during general anesthesia. Previous research has suggested that the administration of remimazolam prior to the end of surgery can reduce the incidence of ED in pediatric patients [18]. However, the extent to which remimazolam may prevent the occurrence of ED relative to dexmedetomidine remains uncertain.

The objective of this study was to assess and compare the incidence and severity of ED in pediatric patients who received a single dose of either remimazolam or dexmedetomidine before the end of sevoflurane anesthesia during tonsillectomy and adenoidectomy surgery.

## 2. Methods

### 2.1. Study Design

**Figure 1:**
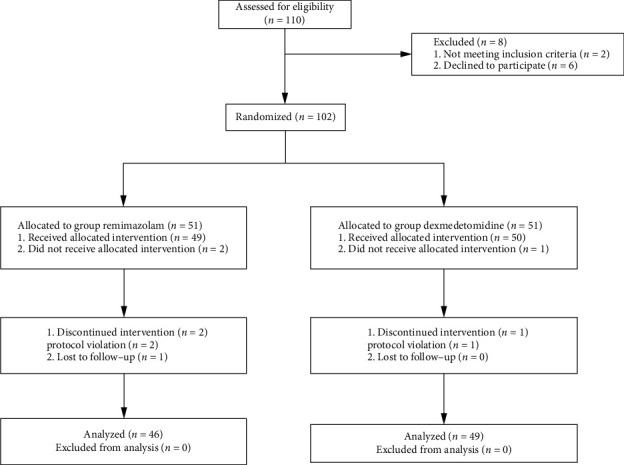
CONSORT flow diagram of the patients included in the study.

This trial was conducted as a single-center, double-blind, and prospective randomized clinical trial with double-arm parallel grouping to compare the efficacy of remimazolam versus dexmedetomidine for the prevention of ED in children under sevoflurane anesthesia. This study was approved by the Ethics Committee of Tongji Hospital of Huazhong University of Science and Technology (TJ-IRB20230508), and written informed consent was obtained from all subjects participating in the trial. The study flowchart is depicted in Figure 1.

### 2.2. Patients

The inclusion criteria for this study were defined as follows: [1] age range of 2–12 years, [2] American Society of Anesthesiologists (ASA) Classification I or II, and [3] patients who underwent elective tonsillectomy and/or adenoidectomy under sevoflurane anesthesia.

The exclusion criteria for this study were as follows: (1) age > 12 years or age < 2 years; (2) ASA Classification ≥ III; (3) known allergy to dexmedetomidine or remimazolam; (4) preoperative pulmonary dysfunction, such as pneumonia, acute lung injury, and acute respiratory distress syndrome; (5) preoperative cardiac dysfunction; (6) preoperative liver or kidney dysfunction; (7) history of mental illness; (8) presence of developmental delays or neurological disorders; and (9) participation in other trails within the last 90 days.

### 2.3. Randomization and Intervention

To ensure unbiased group allocation, an independent investigator, who is not engaged in anesthetic management or postoperative follow-up, utilized SPSS V.27.0 to generate a randomized number table. This randomization process divided the patients into two groups, namely, the dexmedetomidine group (D group) and the remimazolam group (R group), in a 1:1 ratio. The random numbers were sealed within opaque envelopes. The study drugs, contained in identical syringes, were prepared by an independent research anesthesiologist who opened the envelopes before the surgery. This physician was not engaged in patient care or data collection. The group allocations were hidden from the parents of each participant, the attending anesthesiologists, the compassionate nurses, and the study investigators.

Each patient was not given any medicine beforehand, and they were all escorted into the operation room by a parent. There, a general anesthetist who was blind to the patients' group assignment administered anesthesia. Upon arrival in the operation room, the patient's vital signs, including electrocardiogram (ECG), noninvasive arterial blood pressure (NIBP), and pulse oxygen saturation (SpO_2_), were regularly monitored at 3-min intervals. During this period, an anesthesiologist not involved in the study design assessed the patient's condition based on the Pediatric Anesthesia Behavior score (PABs) [19] (Supporting Table 1). The anesthesia induction involved the administration of propofol at a dosage of 2–3 mg/kg, sufentanil at a dosage of 0.2–0.5 μg/kg, and cisatracurium at a dosage of 0.05–0.1 mg/kg. In addition, dexamethasone and penehyclidine hydrochloride were administered to enhance the quality of anesthesia. Simultaneously, all pediatric patients were preoxygenated for a few minutes in 100% oxygen at a flow rate of 6 L/min, aiming to increase their tolerance to hypoxia during intubation. When the pediatric patient's eyelash faded, and there was no response to shoulder tapping, a tracheal tube of adequate diameter was inserted under the guidance of a visual laryngoscope to secure the airway.

The specific settings were adjusted based on the patient's age and ideal body weight, aiming to maintain end-tidal carbon dioxide (Pet-CO_2_) levels between 35 and 45 mmHg. Respectively, remifentanil and sevoflurane were continuously infused at rates of 0.1–0.3 μg/(kg•min) and 2%-3% to maintain anesthesia. Sevoflurane inhalation was discontinued 15 min prior to the conclusion of the surgical procedure, a decision made in consultation with the surgeon regarding the procedure's status. Simultaneously, the participants in the study were randomly assigned to one of two groups: the R group, consisting of 51 children who received intravenous remimazolam at a dosage of 0.2 mg/kg, or the D group, consisting of 51 children who received a single dose of dexmedetomidine at a dosage of 0.2 μg/kg over 2 min to minimize its hemodynamic impact as a manufacture recommendation of 1 μg/kg within 10 min. In addition, in order to reduce the potential influence of pain on the research outcomes, each child was administered a concurrent intravenous injection of 0.05 μg/kg of sufentanil. The endotracheal tube was withdrawn while the pediatric patients were under deep anesthesia, and the patients were transferred to the PACU, once their spontaneous respiratory function and airway reflexes had returned. If ED occurred, 1 mg/kg of propofol was administered as a rescue medication and administered again if the delirium persisted.

### 2.4. Outcome Assessments

The primary focus of the study was to assess the incidence of ED. The Pediatric Anesthesia ED (PAED) scale [20] (Supporting Table 2) was employed to assess ED at 5 min intervals throughout the initial 30 min in the PACU. Scores of 10 or more were used to diagnose ED, and scores of more than 15 were used to describe serious ED. If the child spent less than half an hour in the PACU, PAED scores were documented every 5 minutes until discharge from the recovery room.

Secondary outcomes included the peak PAED scores, Richmond Agitation and Sedation Scale (RASS) [21] (Supporting Table 3) when extubating, time to spontaneous breathing recovery (defined as the duration from intervention administration to the resumption of spontaneous breathing), extubation time (defined as the time interval between intervention administration and the removal of the endotracheal tube), the length of PACU stay (defined as the time from extubation to discharge from the PACU), parental satisfaction, and postoperative nausea and vomiting (PONV). The parental satisfaction score, assessed during the 24-h postoperative follow-up, ranged from 1 (*least satisfied*) to 10 (*most satisfied*) as reported by the children's parents.

Furthermore, the study included the monitoring of vital signs, including heart rate (HR), systolic blood pressure (SBP), and diastolic blood pressure (DBP), with measurements taken at the following time points: immediately before administration of the study drug (baseline, T_0_); 1 min (T_1_), 3 min (T_2_), 5 min (T_3_), and 10 min (T_4_) after administration; and at the time of extubation (T_5_) and 3 min (T_6_) after extubation. The research also reported postoperative adverse events, including bradycardia (defined as a HR below 50 beats per minute), oxygen desaturation (defined as peripheral capillary oxygen saturation below 90%), and laryngospasm. These parameters were systematically assessed to comprehensively evaluate the safety and efficacy of the interventions under investigation.

### 2.5. Statistical Analysis

Following dexmedetomidine intervention, the incidence of ED in children was approximately 36% [22]. To achieve a statistical power of 90% and an α error of 0.05, a sample size of 50 patients per group was determined as necessary for detecting a 25% reduction in the incidence of ED. To account for a 10% drop rate, we needed a total of 110 patients.

Continuous variables such as demographic characteristics (age, height, weight, and BMI) and length of stay in the PACU were presented as mean ± standard deviation (SD) or median (25th–75th IQR). Continuous variables were analyzed by the independent *t*-test or Mann–Whitney *U* test. Categorical variables such as incidence of ED and incidence of adverse events were presented as percentages and compared by Fisher's exact test and *χ*^2^ test, and *p* < 0.05 was considered statistically significant. If the proportion of lost-to-follow-up cases exceeded 5% of the total projected sample size, the multiple imputation would be employed to maintain statistical power by compensating for missing data.

In addition, logistic regression analysis was employed to construct a regression model, aiming to identify the risk factors associated with ED and assess the respective magnitudes of these factors. The statistical analyses and graphs were performed with the GraphPad Prism 9.0 and SPSS 27.0 software.

## 3. Results

Figure 1 displays a CONSORT flow diagram that outlines the process of subject eligibility, randomization, and follow-up. A total of 110 patients were enrolled in the study, of whom 2 were found ineligible after admission and excluded, and 6 patients withdrew temporarily. In addition, 3 patients withdrew because the procedure took longer than planned due to extended intraoperative hemostasis times, 3 patients were excluded due to dexmedetomidine being used during induction, and 1 patient was excluded due to postoperative loss to follow-up. Therefore, just 95 patients were qualified for the final statistical analysis. We performed an intention-to-treat analysis on the data since the missingness rate was more than 5%. We used the multiple imputation to impute missing data.

Intention-to-treat analysis indicated that the mean age of patients was 5.7 ± 0.4 years, with 62 (56.4%) of them being male. The demographic variables, including age, sex, BMI, and ASA status, as well as the perioperative clinical data, such as vital signs at arrival, PABs, duration of surgery, anesthesia duration, and kind of surgery, were found to be similar between the two groups, as shown in Table 1.

**Figure 2:**
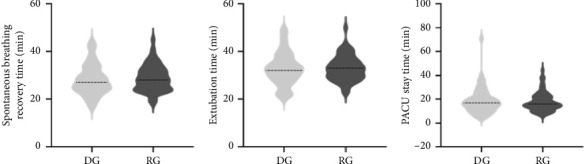
The recovery time of (a) spontaneous breathing, (b) extubation time, or (c) duration of stay in the PACU in children receiving dexmedetomidine (D G) and remimazolam (R G).

**Figure 3:**
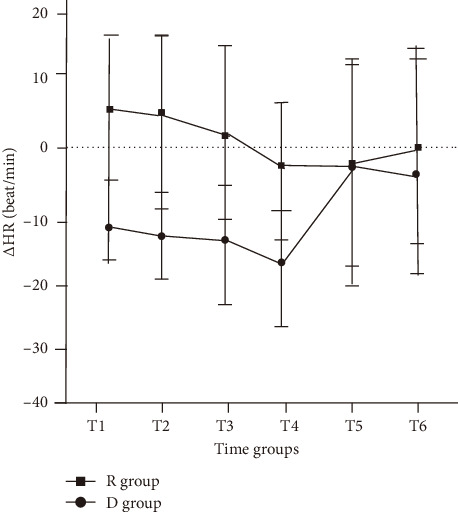
The line chart of heart rate in children receiving dexmedetomidine (D group) and remimazolam (R group). ^∗∗∗^*p* < 0.001.

**Figure 4:**
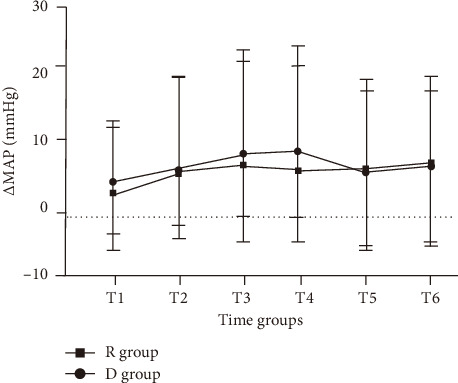
The line chart of the mean blood pressure in children receiving dexmedetomidine (D group) and remimazolam (R group).

Among the patients who received actual intervention, the incidence of ED in the R group was 17.4% (8/46), while in the D group, it was 22.4% (11/49). There was no significant difference between the two groups (*p*=0.538). In addition, the intention-to-treat analysis showed the incidence of ED was found to be 25.5% overall, with no significant difference seen between the two groups (Table 2). Most cases of ED were self-limiting and resolved within 15 min. The peak PAED scores were similar in the R group and D group (13.6 ± 3.1 vs 13.8 ± 1.1, *p*=0.651).  Figure 2 indicates that there were no statistically significant differences seen among the groups in relation to the recovery time of spontaneous breathing, extubation time, or duration of stay in the PACU. Six (10.9%) of remimazolam patients and four (7.3%) of dexmedetomidine patients observed adverse events (Table 2). There were no differences in the incidence of adverse events, or the proportions of patients requiring interventions between the two groups. In terms of parental satisfaction and PONV, there were no significant differences between the two groups. Interestingly, as shown in Figure 3, we did find that HR dropped significantly after dexmedetomidine administration compared to the R group, and this reduction persisted for at least 10 min (*p* < 0.001). As shown in Figure 4, there was no statistically significant difference in the mean arterial pressure change between the R group and the D group of patients (*p* > 0.05). Changes in blood pressure and HR at each specific time point are shown in Supporting Table 4.

**Figure 5:**
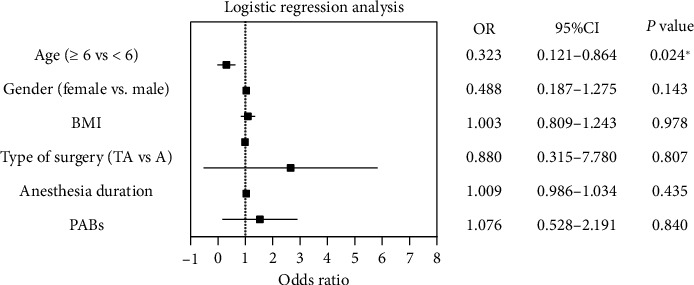
Forest plot of predictors of emergence delirium. ^∗^*p* < 0.05.

A binary logistic regression analysis was conducted to identify the predictors of ED among the recorded variables, which included age, sex, BMI, length of anesthesia, kind of surgery, and the PABs. When age was used as a continuous variable, the *p* value in logistic regression was 0.069. However, as depicted in Figure 5, when age was used as a categorical variable, it was the only significant predictor of ED (*p* < 0.05, OR = 0.323, 95% CI 0.121–0.864). Thus, compared to preschoolers, school-age children have a 32% lower chance of having ED.

## 4. Discussion

In this randomized trial, we compared the efficacy of remimazolam versus dexmedetomidine in preventing ED in children. In the primary analysis, which included patients eligible for the protocol population, and in the intention-to-treat analysis, we observed that the incidence and severity of ED were similar in both groups, with no statistical significance. Moreover, there were no statistically significant differences in postoperative adverse events, length of stay in the PACU, or parental satisfaction between the two groups. In relation to the level of severity, it was observed that children in the D group had more severe episodes of emerging delirium. This is evident from the fact that two patients in this group required pharmacological intervention, whereas no children in the R group necessitated such therapy. However, the observed differences did not reach statistical significance.

Previous studies have indicated that postoperative pain [10] and perioperative anxiety [23] are risk factors for ED. To mitigate the potential impact of pain on the study outcomes, we administered a dose of 0.05 μg/kg of sufentanil at the time of intervention, thereby excluding pain-related considerations from the evaluation of the results. Sufentanil's high lipid solubility and rapid onset of intravenous action make it the perfect choice for analgesic transitions before the end of surgery [24]. As a result, the probability of pain acting as a confounding factor was reduced. In this clinical study, children in the D group and R group exhibited comparable preoperative anxiety scores. Contrary to what was shown in earlier trials [25, 26], there was no statistically significant link between preoperative anxiety and ED. The varied application of scales to evaluate preoperative anxiety levels and the presence of parents [27] in the preoperative waiting area may be the reason for the inconsistent results.

The administration of dexmedetomidine may result in a reduction in HR [28]. To minimize the effect of reducing HR, we administered the dose with a 20 mL syringe and delivered a bolus dose over at least 2 min. However, in our current investigation, HR was considerably lower in the D group than in the R group for 10 min following the study medication given. This could be attributed to the activation of presynaptic *α* receptors, inhibition of norepinephrine release, and sympathetic conduction when dexmedetomidine is administered slowly at low doses, resulting in a decrease in HR [29]. Thus, remimazolam has a lesser hemodynamic impact and induces fewer changes in HR compared to dexmedetomidine, which is similar to the previous study [14, 30].

The mechanism of ED is uncertain; however, it may be related to rapid awakening. As a result, we established this clinical research to include an intravenous sedative as a bridge to the end of sevoflurane anesthesia, which could be useful in sevoflurane washout before emergence. Remimazolam exhibited similar clinical properties to dexmedetomidine in this trial, suggesting that remimazolam could serve as a viable alternative adjunct for preventing ED.

The final number of patients completing the study was 95, with a dropout rate of 13.6%, making it unsuitable to exclude missing data to use full data analysis [31].

There are some limitations related to the study. First, we did not include a saline group as a blank control, which could have provided a more comprehensive comparison. Second, our conclusions were based on a small sample size using a single dose in a single center, which is a preliminary study. In addition, the number of patients included in the final statistical analysis was less than the initially planned quantity, and statistical efficacy was only 75% in the post hoc analysis. To compensate for the impact of missing sample size on efficacy, we employed intention-to-treat analysis. However, conducting multicenter, large-sample clinical investigations would be necessary to further validate our findings. Third, because the period of follow-up was restricted to 1 day, the long-term efficacy of remimazolam in preventing emerging delirium is unknown. In addition, to maintain blinding integrity, both remimazolam and dexmedetomidine were administered 15 min before the end of surgery. However, as an ultrashort-acting benzodiazepine, this administration timing may have attenuated remimazolam's efficacy in preventing ED.

In summary, for children undergoing sevoflurane anesthesia, administration of 0.2 mg/kg of remimazolam before the end of surgery did not show superiority over dexmedetomidine in preventing ED. Remimazolam may have an advantage over midazolam in accelerating extubation or reducing PACU stay. The only predictor of ED is age, and the incidence of delirium decreased by 32% for every 6 years of age increase.

## Figures and Tables

**Table 1:** Demographic and clinical characteristics of the patient population.

	Dexmedetomidine group (*n* = 55)	Remimazolam group (*n* = 55)	*p* value
Age (years)	5.6 ± 1.6	5.7 ± 0.6	0.665
0–6	31 (56.4)	29 (52.7)	
≥ 6	24 (43.6)	26 (47.3)	0.702
Sex; *n* (%)			
Male	32 (58.2)	30 (54.5)	
Female	23 (41.8)	25 (45.5)	0.700
BMI (kg m^2 −1^)	15.4 ± 1.1	15.5 ± 0.7	0.570
Important vital signs upon entry			
MAP (mmHg)	75.4 ± 11.2	77.6 ± 13.2	0.348
HR (beat min^−1^)	92.2 ± 4.8	90.3 ± 8.9	0.659
S_P_O_2_ (%)	99.0 [98.0 to 100.0]	99.0 [98.0 to 100.0]	0.910
Anesthesia duration (min)	38.0 [28.0 to 57.0]	34.0 [27.0 to 58.0]	0.140
Surgery duration (min)	44.0 [27.0 to 43.0]	41.0 [33.0 to 54.0]	0.639
ASA physical status; *n* (%)			
1	55 (100)	55 (100)	/
Pediatric anesthesia behavior score; *n* (%)			
1	51 (92.7)	48 (87.3)	
3	4 (7.3)	7 (12.7)	0.340
Type of surgery; *n* (%)			
Tonsillectomy and adenoidectomy	31 (56.4)	39 (70.9)	
Adenoidectomy	24 (43.6)	16 (29.1)	0.113

*Note:* The duration of administration is defined as the time between administration and the end of surgery.

Abbreviations: ASA, American Society of Anesthesiologists; BMI, body mass index.

**Table 2:** The primary and secondary outcomes of the study.

	Dexmedetomidine group (*n* = 55)	Remimazolam group (*n* = 55)	*p* value
Emergence delirium, *n* (%)	11 (20.0)	17 (31.0)	0.189
Peak of PAED score	13.6 ± 3.1	13.8 ± 1.1	0.651
RASS, *n* (%)			
2	2 (3.6)	2 (3.6)	
1	7 (12.7)	4 (7.3)	
0	32 (58.2)	36 (65.5)	
−1	9 (16.4)	8 (14.5)	
−2	5 (9.0)	5 (9.1)	0.671
Spontaneous breathing recovery time (min)	28.2 ± 1.0	28.1 ± 2.3	0.768
Extubation time (min)	32.8 ± 6.9	33.0 ± 5.3	0.119
Length of PACU stay (min)	18.6 ± 11.0	18.0 ± 8.2	0.339
Adverse events, *n* (%)			
Bradycardia	0 (0)	0 (0)	
S_P_O_2_ < 90%	1 (1.8)	3 (5.5)	
S_P_O_2_ < 85%	2 (3.6)	2 (3.6)	
Hypotension	0 (0)	0 (0)	
Laryngospasm	1 (1.8)	1 (1.8)	0.175
Parental satisfaction; *n* (%)			
10	46 (83.6)	42 (76.4)	
9	4 (7.3)	6 (10.9)	
8	4 (7.3)	5 (9.1)	
6	1 (1.8)	2 (3.6)	0.121
PONV, *n* (%)	5 (9.1)	6 (10.9)	0.751

*Note:* PAED, Pediatric Anesthesia Emergence Delirium Scale.

Abbreviations: PACU, postanesthesia care unit; PONV, postoperative nausea and vomiting; RASS, Richmond Agitation Sedation Scale.

## Data Availability

The data are not publicly available due to privacy or ethical restrictions.

## Supporting Information

The scoring criteria for the PABs are provided in Supporting Table 1, those for the PAED scale in Supporting Table 2, and those for the RASS in Supporting Table 3. Changes in vital signs at each time point are presented in Supporting Table 4.
