# Supplementary material for: A Comparison of Single Dose Remimazolam With Dexmedetomidine for the Prevention of Emergence Delirium in Children Undergoing Tonsillectomy and Adenoidectomy Surgery Under Sevoflurane Anesthesia: A Randomized Clinical Trial
Source: Anesthesiol Res Pract. 2025 Sep 14;2025:7780635. doi: 10.1155/anrp/7780635 (PMC12450552; doi:10.1155/anrp/7780635)
Supplement: Supporting Information — Additional supporting information can be found online in the Supporting Information section. [file 7780635.f1.zip › Supplemental Table1.docx]

| **Score** | **Description of behavior** | |
| --- | --- | --- |
| 1 | Happy | Calm and controlled. Compliant with induction |
| 2 | Sad | Tearful and/or withdrawn but compliant with induction |
| 3 | Mad | Loud vocal resistance (screaming or shouting) AND/OR Physical resistance to induction requires physical restraint by staff and/or parents |

Supplemental Table 1 The Pediatric Anesthesia Behavior score
