# Supplementary material for: A Comparison of Single Dose Remimazolam With Dexmedetomidine for the Prevention of Emergence Delirium in Children Undergoing Tonsillectomy and Adenoidectomy Surgery Under Sevoflurane Anesthesia: A Randomized Clinical Trial
Source: Anesthesiol Res Pract. 2025 Sep 14;2025:7780635. doi: 10.1155/anrp/7780635 (PMC12450552; doi:10.1155/anrp/7780635)
Supplement: Supporting Information — Additional supporting information can be found online in the Supporting Information section. [file 7780635.f1.zip › Supplemental Table2.docx]

**Supplemental Table 2** Pediatric anesthesia emergence delirium (PAED) scale

| Behavior | Not at all | Just a little | Quite a bit | Verymuch | Extremely |
| --- | --- | --- | --- | --- | --- |
| Makes eye contact | 4 | 3 | 2 | 1 | 0 |
| Actions are purposeful | 4 | 3 | 2 | 1 | 0 |
| Aware of surroundings | 4 | 3 | 2 | 1 | 0 |
| Restless | 0 | 1 | 2 | 3 | 4 |
| Inconsolable | 0 | 1 | 2 | 3 | 4 |

1—Calm; 2—not calm but could be easily consoled; 3—moderately agitated or restless and not easily calmed; 4—combative, excited, thrashing around.
