# Supplementary material for: A Comparison of Single Dose Remimazolam With Dexmedetomidine for the Prevention of Emergence Delirium in Children Undergoing Tonsillectomy and Adenoidectomy Surgery Under Sevoflurane Anesthesia: A Randomized Clinical Trial
Source: Anesthesiol Res Pract. 2025 Sep 14;2025:7780635. doi: 10.1155/anrp/7780635 (PMC12450552; doi:10.1155/anrp/7780635)
Supplement: Supporting Information — Additional supporting information can be found online in the Supporting Information section. [file 7780635.f1.zip › Supplemental Table3.docx]

| **Score** | **Term** | **Description** |
| --- | --- | --- |
| +4 | Combative | Overtly combative or violent; immediately danger to staff |
| +3 | Very agitated | Pulls on or removes tube(s) or catheter(s) or has aggressive behavior toward staff |
| +2 | Agitated | Frequent no purposeful movement or patient-ventilator desynchrony |
| +1 | Restless | Anxious or apprehensive but movements not aggressive or vigorous |
| 0 | Alert and calm |  |
| -1 | Drowsy | Not fully alert, but has sustained (more than 10 seconds) awakening, with eye contact, to voice |
| -2 | Light sedation | Briefly (less than 10 seconds) awakens with eye contact, to voice |
| -3 | Moderate sedation | Any movement (but no eye contact) to voice |
| -4 | Deep sedation | No response to voice, but any movement to physical stimulation |
| -5 | Unarousable | No response to voice or physical stimulation |

Supplemental Table 3 The Richmond Agitation-Sedation Scale (RASS): a sedation scoring system containing 10 levels with descriptions for each category.
