# Supplementary material for: A Comparison of Single Dose Remimazolam With Dexmedetomidine for the Prevention of Emergence Delirium in Children Undergoing Tonsillectomy and Adenoidectomy Surgery Under Sevoflurane Anesthesia: A Randomized Clinical Trial
Source: Anesthesiol Res Pract. 2025 Sep 14;2025:7780635. doi: 10.1155/anrp/7780635 (PMC12450552; doi:10.1155/anrp/7780635)
Supplement: Supporting Information — Additional supporting information can be found online in the Supporting Information section. [file 7780635.f1.zip › Supplemental Table4.docx]

|  | Group | T_0_ | T_1_ | T_2_ | T_3_ | T_4_ | T_5_ | T_6_ |
| --- | --- | --- | --- | --- | --- | --- | --- | --- |
| HR | R | 92.6±16.4 | 99.8±13.3*** | 98.4±14.5*** | 96.8±13.0*** | 89.9±13.1*** | 91.0±16.2*** | 94.7±16.8*** |
|  | D | 96.7±17.4 | 84.5±15.2*** | 80.8±14.5*** | 79.7±15.0*** | 76.7±13.2*** | 91.3±15.7*** | 92.6±16.0*** |
| SBP | R | 99.8±8.7 | 104.9±11.0*** | 109.4±10.5*** | 111.1±11.1*** | 108.6±11.4*** | 107.4±11.7*** | 108.1±11.6*** |
|  | D | 103.3±14.7 | 105.5±16.1*** | 108.3±16.0*** | 111.7±16.7*** | 111.1±16.6*** | 108.8±16.4*** | 108.4±16.8*** |
| DBP | R | 58.1±11.9 | 63.0±13.9*** | 68.3±13.3*** | 69.5±13.5*** | 68.9±11.8*** | 63.7±12.9 | 65.6±13.3 |
|  | D | 61.4±13.4 | 67.7±2.2*** | 70.9±15.3*** | 73.5±15.8*** | 73.7±15.4*** | 65.1±14.6*** | 65.3±16.5*** |

**Supplemental Table 4** Changes in heart rate and blood pressure of the child at each time point from T_0_ to T_6_. Compared with T_0_, ****P* < 0.001.
